# Is Innervation of the Neuromuscular Junction at the Diaphragm Modulated by sGC/cGMP Signaling?

**DOI:** 10.3389/fphys.2020.00700

**Published:** 2020-06-23

**Authors:** Nadežda Lukáčová, L’udmila Hricová, Alexandra Kisucká, Štefánia Papcunová, Katarína Bimbová, Mária Bačová, Jaroslav Pavel, Martin Marsala, Ivo Vanický, Zuzana Dzurjašková, Stanislav Matéffy, Viktória Lukáčová, Andrea Stropkovská, Ján Gálik

**Affiliations:** ^1^Institute of Neurobiology, Biomedical Research Center of the Slovak Academy of Sciences, Košice, Slovakia; ^2^Neuroregeneration Laboratory, Department of Anesthesiology, University of California, San Diego, La Jolla, CA, United States; ^3^Diagnostic Center of Pathology in Prešov, Alpha Medical, s.r.o., Martin, Slovakia; ^4^Faculty of Economics, Technical University of Košice, Košice, Slovakia

**Keywords:** sGC/cGMP signaling, lower respiratory pathway, rat phrenic nerve ligation, phrenic nerve axonal degeneration, diaphragm neuromuscular junctions

## Abstract

We previously reported NO/sGC signaling in the upper respiratory pathway, receiving input from the respiratory neurons of the brainstem to phrenic motoneurons in the C3–C6 spinal cord. In order to assess whether innervation of the neuromuscular junction (NMJ) at the diaphragm is modulated by sGC/cGMP signaling, we performed unilateral 8-day continuous ligation of the phrenic nerve in rats. We examined sGCβ1 within the lower bulbospinal pathway (phrenic motoneurons, phrenic nerves and NMJs at the diaphragm) and the cGMP level in the contra- and ipsilateral hemidiaphragm. Additionally, we characterized the extent of phrenic nerve axonal degeneration and denervation at diaphragm NMJs. The results of our study show that continuous 8-day phrenic nerve ligation caused a marked increase in sGCβ1 (immunoreactivity and the protein level) in the ipsilateral phrenic motor pool. However, the protein sGCβ1 level in the phrenic nerve below its ligation and the cGMP level in the ipsilateral hemidiaphragm were evidently decreased. Using confocal analysis we discovered a reduction in sGCβ1-IR boutons/synaptic vesicles at the ipsilateral MNJs. These findings are consistent with the marked axonal loss (∼47%) and significant NMJs degeneration in the ipsilateral diaphragm muscle. The remarkable unilateral decrease in cGMP level in the diaphragm and the failure of EMG recordings in the ipsilateral hemidiaphragm muscle can be attributed to the fact that sGC is involved in transmitter release at the diaphragm NMJs via the sGC-cGMP pathway.

## Introduction

The cervical spinal cord is the most common site of traumatic injury leading to interruption of the descending respiratory pathway, which begins in the brainstem, innervates phrenic motoneurons (PhMNs) in the cervical spinal cord, and controls the diaphragm via the phrenic nerves (PhNs) (Mantilla and Sieck, 2008). Hemidiaphragm paralysis due to unilateral phrenic nerve injury is a well-recognized complication in a variety of etiologies, such as cardiac surgery, neck surgery, chiropractic manipulation, and interscalene nerve blocks (Aguirre et al., 2013). Such injuries lead to breathing rhythm abnormalities and life-threatening weakness in respiratory function. Despite significant progress in the development of new post-injury interventions to restore respiratory function after cervical and/or phrenic nerve injuries (Alilain et al., 2011; Sharma et al., 2012; Aguirre et al., 2013) a number of fundamental questions crucial for understanding the signaling in brainstem-diaphragm circuits remain to be further explored.

The nitric oxide/soluble guanylyl cyclase/cyclic guanosine monophosphate (NO/sGC/cGMP) signaling pathway is essential for controlling a number of physiological processes, such as neuronal transmission, cell growth and proliferation (Weill and Greene, 1984; Thippeswamy and Morris, 1997; Fiscus, 2002). Previously, we identified changes in the level of NO-related enzymes in the upper bulbospinal respiratory descending pathway in response to C3 hemisection (Marsala et al., 2002; Capkova et al., 2011). We showed that high cervical spinal cord hemisection followed by 8 days of survival caused strong depletion of neuronal nitric oxide synthase (nNOS) fluorescent terminals around the sGCβ1 subunit immunoreactive (sGCβ1-IR) phrenic motoneurons on the side of the injury. In addition, sGCβ1-IR was significantly reduced in the contralateral and nearly eliminated in ipsilateral PhMNs (Capkova et al., 2011). These data indicate that the guanylyl cyclase phosphorylation cascade, which is activated by NO in the brainstem, could be localized in motor nerve terminals. Several previous studies have indicated that nNOS, cGMP and cGMP-dependent kinase are all concentrated at or near the neuromuscular junction (NMJ) (Kobzik et al., 1994; Chao et al., 1997). Herring and Paterson (2001) demonstrated that NO stimulates presynaptic sGC to produce cGMP at the vagal-atrial junction in guinea-pigs. These data are consistent with the findings of Nickels et al. (2007) showing that NO facilitates transmitter release *in vivo* by way of a cGMP- and cAMP-mediated mechanism involving the activation of N-type Ca^2+^ channels.

The compensatory mechanisms occurring following unilateral diaphragm hemiparalysis are not fully elucidated. Nicaise et al. (2013) demonstrated the early time course of phrenic motor neuron degeneration, persistent phrenic nerve axonal degeneration, and consequent respiratory deficits following unilateral cervical spinal cord contusion. These authors reported that the diaphragm compound muscle action potential amplitudes were first reduced at 24 h after C4 contusion (30% of pre-injury maximum amplitude) and afterward some slow functional improvement associated with partial reinnervation at the diaphragm NMJ was seen at 8 and 14 days post-injury. Furthermore, complete phrenic nerve inactivation and ipsilateral paralysis were detected after a lateral area section of the C2 cervical spinal cord (Vinit et al., 2006). Although the activity of the ipsilateral PhN was partially restored after a lapse of 3 months, no spontaneous diaphragm recovery was observed, even after several months. Additionally, Gill et al. (2015) reported an increase in central respiratory drive after acute phrenic nerve denervation. These authors showed that a compensatory loading effect on the contralateral diaphragm may contribute to an increase in central drive to contralateral phrenic motor neurons.

Although NO-sGC signaling has been established in the brainstem-spinal cord circuitry, there have been no investigations into whether signaling in the lower bulbospinal respiratory pathway (phrenic motoneurons – phrenic nerve – diaphragm) is modulated by sGC-cGMP induced mechanisms. We therefore studied this signaling in a rat model of unilateral phrenic nerve ligation. We tested the hypothesis that PhN ligation-induced decrease in sGCβ1-IR terminal boutons at diaphragm NMJs could affect cGMP-dependent formation in the diaphragm. In order to verify the absence of ipsilateral diaphragm activity and thus the completeness of the phrenic nerve injury, we combined this analysis with diaphragm EMG activity measured immediately after phrenic nerve injury and again on the eighth day, when the experiments were terminated.

## Materials and Methods

### Experimental Animals

Experiments were performed with a total of 50 adult male Wistar rats weighing 300–450 g. The animals were divided into three experimental groups: (1) sham-operated animals (*n* = 20), (2) rats subjected to unilateral PhN ligation followed by 8 days of survival (*n* = 25), and (3) rats subjected to unilateral PhN ligation followed by bilateral injection of retrograde tracer Fluorogold (FG) into the diaphragm on the sixth day; 8 days survival of animals (*n* = 5). All surgical procedures and post-operative animal care were approved by the Animal Care Committee at the Institute of Neurobiology, Slovak Academy of Sciences. The experiments conformed to the National Institutes of Health Guide for the Care and Use of Laboratory Animals. The experimental animals were housed in individual cages and given food and water ad libitum. The rats were kept in a 12 h light/dark cycle at a temperature of 23°C.

### Ligation of Phrenic Nerve

The animals were deeply anesthetized with isoflurane (Abbott, Queenborough, United Kingdom; in 1.5–2.0 L/min oxygen) and ventilated in a respirator with oxygen and nitrous oxide (1:1). Afterward, the rats were rested in supine position on the operating table. The body temperature was maintained at 37°C during the whole surgical procedure. Under aseptic conditions, a midline incision was made through the neck skin and muscles of the left sixth intercostal space. The left phrenic nerve was elevated with a hook and two ligatures were tied around nerve with approx. 0.5 cm distance inbetween. The PhN ligation was performed uniformly using a solid surgical suture (sterile, silk thread, 1C, Leciva Praha, Czech Republic) without disrupting the nerve continuity and was confirmed by identifying the changes in abdominal and rib cage movements associated with breathing. Once the movements in the ipsilateral hemidiaphragm completely disappeared (checking by palpation), the muscles and skin were sutured with silk. The rats received Amoksiklav antibiotic (Sandoz Pharmaceuticals, Ljubljana, Slovenia; 30 mg/kg, i.m.) and Novasul analgesic (Richterpharma, Wels, Austria; 2 ml/kg, i.m.) for 3 days. The animals were housed in separated cages to recover with access to food and water ad libitum and they survived for 8 days (*n* = 25). In the sham-operated group the animals were subjected to a midline incision through the neck skin and muscles of the left sixth intercostal space without ligation of the phrenic nerve (*n* = 20).

### Ligation of Phrenic Nerve and Retrograde Tracing

Retrograde labeling with Fluorogold fluorescent tracer (Hydroxystilbamidine, methanesulfonate, BioChemika, Steinheim, Germany) was used for visualization of the sGCß1-IR in a pre-labeled phrenic motor neurons of C3-C6 segments. Six days after unilateral PhN ligation the animals (*n* = 5) were deeply re-anesthetized with isoflurane as described above. An abdominal incision was made to expose the whole diaphragm muscle. Fluorogold (FG; 4% solution dissolved in 0.9 saline) was injected bilaterally into the diaphragm along the primary branches of the phrenic nerves (six injection sites of 3 μl of each) using a 10 μl Hamilton syringe. The abdominal muscles and the skin were sutured, the animals received Novasul (2 ml/kg, i.m.) and Amoksiklav (30 mg/kg, i.m.) and survived for next 2 days.

### sGCβ1 Immunohistochemistry

For immunohistochemical analysis of retrogradely labeled phrenic motoneurons, the animals (*n* = 5) were deeply anesthetized with thiopental (Valeant Czech Pharma s.r.o., Prague, Czech Republic; 50 mg/kg, i.p.) and perfused transcardially through the aorta with 300 ml saline (0.9% NaCl) followed by 300 ml fresh 4% paraformaldehyde in 0.1M phosphate-buffered saline (PBS; pH 7.4, Sigma–Aldrich, St. Louis, MO, United States). The spinal cord segments (C3-C6) were dissected out and postfixed in 4% paraformaldehyde for 3 h, followed by overnight cryoprotection in a solution of 30% sucrose in PBS at 4°C for at least 48 h. Using a cryostat (Leica, CM1850, Wetzlar, Germany), 30μm-thick transverse sections (each fourth slice was collected and used for staining) were cut from the cervical segments (*n* = 5) and used for sGCß1 immunohistochemistry. Free-floating sections of C3-C6 spinal cord segments were pretreated with 0.1 M phosphate-buffered saline (PBS; pH 7.4) for 30 min and blocked for unspecific staining using 5% normal goat serum (NGS) in PBST for 2 h (PBS with 0.4% Triton). Then the sections were transferred into rabbit polyclonal antibody to Guanylyl Cyclase (β1) (1:100, ab24824, Abcam, Cambridge, United Kingdom) in PBST plus 5% NGS at 4°C for 48 h. After rinsing with PBS, the sections were incubated with goat anti-rabbit IgG (1:200, 111-295-144, Jackson ImmunoResearch, Baltimore, MD, United States) for 1 h at room temperature. Sections were washed 4 × 5 min with PBS, mounted on glass slides, air-dried overnight, cleared with xylene and cover-slipped with Fluoromont (21644, Serva, Heidelberg, Germany). Negative controls were prepared by omitting the sGCß1 primary antibody. Images were captured using an Olympus BX51 (Tokyo, Japan) fluorescent microscope.

### Counting of Phrenic Motoneurons

First, the retrogradely labeled phrenic nucleus was visually inspected, clearly identified on the contralateral side and analogously also on the side of PhN ligation. The sGCβ1- positive phrenic motoneurons were individually identified and counted manually from 60 randomly taken FG-labeled/sGCβ1- immunofluorescently stained sections (12 slices/rat). Only phrenic motoneurons showing cytoplasmic staining were counted. The cells were counted by two investigators independently in blind manner, and expressed as number of PhMNs/slide.

### sGCβ1 and Neurofilament (NF) Immunohistochemistry

After transcardial perfusion (see above), the control phrenic nerves (*n* = 5) and the phrenic nerves after ligation (*n* = 5) were dissected out and postfixed in 4% paraformaldehyde for 3 h, followed by overnight cryoprotection in a solution of 30% sucrose in 0.1M PBS at 4°C for at least 48 h. Using a cryostat (Leica, Nussloch, Germany), 20 μm-thick longitudinal sections (taken from the phrenic nerves below their ligation) were cut, stretched on superfrost slides and used for double immunostaining. Incubation with sGCβ1 antibody was the same as for the cervical segments. Phrenic nerves were double-labeled with NF antibody. Before incubation the sections were rinsed for 20 min in PBST and blocked with NGS for 2 h and incubated with Anti-200 kD Neurofilament Heavy antibody (ab7795, Abcam, Cambridge, United Kingdom, dilution 1:500) for 24 h. Afterward, the sections were incubated with goat anti-mouse IgG (1:125 in NGST, 111-295-144, Jackson ImmunoResearch, Baltimore, MD, United States). Tissues were rinsed in PBS for 30 min and then coverslipped with Fluoromont.

### Western Blotting

The control rats (*n* = 4) and rats subjected to PhN ligation (*n* = 4) were euthanized on the eighth day of the experiment. Samples from the ventral horn of C3-C6 segments and phrenic nerves were quickly removed from the rats, washed in ice-cold isotonic saline and frozen in liquid nitrogen. Tissues were stored at -80°C until further processing. Phrenic nerves were ground to a powder, then all samples were homogenized in ice-cold homogenization buffer (1 M Tris-HCl, pH 6.8) containing protease inhibitors (Roche, Mannheim, Germany) and then centrifuged at 15 000 × g for 15 min at 4°C. The supernatants were collected and protein content was determined by means of Bradford protein assay (Bradford, 1976). Samples were cooled on ice during the whole procedure. A total of 50 μg protein from each sample was mixed with an equal volume of sample buffer (62.5 mM Tris–HCl, 2% SDS, 100 mM DTT, 0.2 mM 2-mercaptoethanol, 20% glycerol, and 0.5% bromophenol blue, pH 6.8) and denaturated for 5 min at 95°C. Proteins were separated with SDS-PAGE (12%) at a constant voltage of 100 V and transferred to a nitrocellulose membrane (Bio-Rad, Hercules, CA 94547, United States) using the “semi-dry” method of Western blotting (50 min). Precision plus protein all blue standard (Bio-Rad, Hercules, CA 94547, United States) was used to determine approximate molecular weights. Ponceau S (Merck, Sigma, Aldrich, Darmstadt, Germany) was used on each membrane to verify protein transfer. The membranes were washed with distilled water 3 × 5 min and blocked with 5% milk powder (non-fat dry milk) in Tris-buffered saline (TBS) containing 0.05% Tween 20 (TBS-T) for 1 h at room temperature. Then the membranes were incubated with rabbit polyclonal antibody to Guanylyl Cyclase (β1) (1:3000, 160897, Cayman Chemical, Ann Arbor, MI, United States) in TBS-T overnight at 4°C on an orbital shaker. Negative controls were prepared by omitting the sGCß1 primary antibody. The next morning the membranes were washed 4 × 5 min with TBS-T and then incubated with peroxidase-conjugated anti-rabbit secondary antibody (1:20,000, 111-035,003, Jackson Immunoresearch, Baltimore, MD, United States) for 2 h at room temperature. After 4 x 5 min washing with TBS-T, the membranes were incubated using the ChemiLucent Detection System kit (Chemicon International, Temecula, CA 92590, United States) exposed to Hyperfilm ECL (Cytiva, Marlborough, MA, United States). After band visualization, the membranes were washed with Restore Plus Western Blot Stripping Buffer (Thermo Scientific, Waltham, MA, United States) and re-used for incubation with β-actin monoclonal antibody (Merck, Sigma Aldrich, Darmstadt, Germany) at 1:50,000 dilution, which was used as sample loading control and normalization protein as well. We used the Gel Doc XR system (Bio-Rad, Hercules, CA 94547, United States) for scanning of protein bands and quantification was performed using Quantity One software (Bio-Rad, Hercules, CA 94547, United States).

### cGMP Measurement Using ELISA

An ELISA kit was used for determining the level of cGMP in the control diaphragm (*n* = 5) and in hemidiaphragms after the phrenic nerve injury (*n* = 5). The rats were reanesthetized with isoflurane and decapitated, the whole hemidiaphragms were dissected out, frozen in liquid nitrogen and prepared for additional use. Samples were ground to a powder. After the liquid nitrogen evaporated, frozen tissue (300 μg) was homogenized with 0.1 M HCl. A cGMP Enzyme Immunoassay Kit (CG200, Merck, Sigma Aldrich, Darmstadt, Germany) was used for measuring cGMP concentrations in the muscle homogenates. All samples were acetylated (because acetylation of the samples increases the sensitivity of the assay) by adding 10 μl of acetylation reagent (acetic anhydride and triethylamine), and the samples were treated with hydrochloric acid to stop endogenous phosphodiesterase activity. cGMP standards were prepared according to protocol, and the concentrations of cGMP in tubes 1–5 were 50, 10, 2, 0.4, and 0.08 pmol/ml. Tissue samples were centrifuged at 600 × g at room temperature and supernatants were then diluted with 0.1 M HCl. Samples and standards were added in volumes of 100 μl into the appropriate wells (run in duplicates) and then incubated with yellow cGMP EIA antibody at room temperature for 2 h. Thereafter the wells were washed with Wash Buffer, and blue cGMP-Alkaline Phosphatase Conjugate and p-Nitrophenyl Phosphate Substrate Solution were added for 1 h incubation without shaking. The reaction was stopped by adding Stop Solution to every well and the plate was read immediately on a multiwell plate reader at 405 nm, preferably with correction between 570 and 590 nm. The concentration of cGMP was calculated using Logit-Log paper, where we put the percentage bound (optical density of samples and standards) vs. concentration of cGMP for the standards.

### Diaphragm and Phrenic Nerve Preparation for Immunohistochemistry

Eight days after PhN ligation the animals (*n* = 5) were deeply reanesthetized with thiopental (Valeant Czech Pharma s.r.o., Prague, Czech Republic; 50 mg/kg, i.p.), and fresh diaphragm muscle and phrenic nerves were removed in the following steps. Laparotomy was carried out in order to visualize the sternum and xiphoid process. Fat and connective tissue were gently removed using forceps and scissors. The sternum was transversally cut along the ribs to expose the diaphragm and phrenic nerves. Diaphragm muscles and both ipsilateral and contralateral phrenic nerves were carefully dissected out and washed in saline. The dissected diaphragm was pinned down (using insect pins) to silicone-based material (Duosil Expres, SHERA, Lemförde, Germany) in a 90 mm Petri dish to stretch and fix the diaphragm. The diaphragm was afterward postfixed in 4% paraformaldehyde for 15 min. Following postfixation the superior surface of the diaphragm membrane was precisely removed with small forceps. The fixative pins were removed and diaphragm muscles were separated to contralateral and ipsilateral sides and placed in saline to prepare the samples for fluorescence labeling. The phrenic nerves were used for histological staining.

### Immunohistochemical Staining of Diaphragm and Phrenic Nerve

Hemidiaphragms were washed in 0.1 M sodium phosphate buffer (PBS, pH = 7.4) 3 × 10 min. Diaphragm samples were incubated in 0.1 M glycine for 30 min. To visualize post-synaptic acetylcholine receptors of the motor endplate, the diaphragm was labeled with rhodamine-conjugated α bungarotoxin (red, 1:400, Biotium, Fremont, CA 94538, United States) for 15 min. After incubation the samples were washed in PBS (3 × 15 min) and incubated with methanol for 5 min at -21°C. Afterward, the hemidiaphragms were washed again (PBS, 3 × 15 min) and blocked in NGST (0.2% PBST+2% NGS) at room temperature. For visualization of sGCβ1 nerve terminals, synaptic vesicles and motor axons, the samples were incubated with primary antibodies (rabbit, 1:200, Abcam, Cambridge, United Kingdom; SV2, rabbit, 1:200, Cell Signaling, Danvers, MA, United States; SMI-312, chicken 1:1000, Abcam, Cambridge, United Kingdom) overnight at 4°C. Subsequently, the diaphragms were washed in NGST 3 × 10 min and incubated with secondary antibodies (green goat anti-rabbit IgG, 1:250, Jackson Immunoresearch, Baltimore, MD, United States; blue goat anti-chicken IgG, 1:500, Abcam, Cambridge, United Kingdom) at room temperature for 1 h. The diaphragm muscles were finally washed with PBS (3 × 10 min), placed on glass slides and mounted with aqueous medium (Fluoromont, Merck, Sigma Aldrich, Darmstadt, Germany). Images were captured with a confocal microscope (Leica DM 2500, Wetzlar, Germany) using LASAF software. The number of neuromuscular junctions (NMJs) was evaluated using ImageJ 1.47 software (NIH, Bethesda, MD, United States). NMJs were counted in the central region of the contra- and ipsilateral hemidiaphragms and were assessed as intact, partially or fully denervated.

### Phrenic Nerve Staining and Quantitative Analysis

Both phrenic nerves (contralateral and ipsilateral), isolated under thiopental anesthesia, were immersion-fixed in freshly prepared 2.5% glutaraldehyde in 0.1M phosphate buffer at 4°C for 24 h. After fixation, the nerves were stretched and embedded into protein matrix (Bredt et al., 1990). The matrix containing the nerve was placed into a metallic Tissue matrix (TM-1000 10 × 10 mm chamber w/1 mm slices, ASI Instruments, Warren, MI, United States), and cut with a microtome blade into 1 mm thick slabs. The flat slabs allow proper orientation of the nerve tissue in the mold before the resin hardens. The slabs were then postfixed in 1% osmium tetroxide in 0.1M phosphate buffer and embedded into Durcupan according to standard protocol. Transverse sections were cut on a standard sliding microtome (Leica SM2010R, Nussloch, Germany) with special blades for hard materials (Leica 819, Nussloch, Germany). Under a stereomicroscope, 1–3 μm thick sections were teased onto gelatinized slides from 96% ethanol, dried and coverslipped with Polymount. Selected sections were stained with Toluidine Blue. These sections were photographed at 400× magnification. Ten images from each nerve specimen were analyzed using the freely available Neurocounter morphometric software^1^ by two researchers blind to nerve identity to count the number of myelinated axons. This software recognizes myelinized axons as objects and automatically evaluates their number as well as several morphometric parameters.

### Electrophysiology

To assess diaphragm activity, the diaphragm EMG was recorded in controls (*n* = 6), immediately after PhN ligation (*n* = 3) and again later, 8 days after the ligation (*n* = 3). The animals were anesthetized by means of intraperitoneal injection of a combination anesthetic (containing ketamine and xylazine) at a total dose of 1.4–1.8 ml/kg/animal. After laparotomy, the liver was gently pushed away to expose the diaphragm muscle. Bipolar stainless steel electrodes in the form of small parallel hooks (distance of 3 mm) were inserted into the diaphragm muscle. Signals were recorded from both the contralateral and ispilateral hemidiaphragms. The EMG signal was fed into the preamplifier of a differential A/C amplifier (DP 311, Warner Instrument, Hamden, CT, United States), where it was filtered with high-pass (100 Hz) and low-pass (10 kHz). The amplified signal was then sampled at a sampling rate of 20 k/s (PowerLab 8/35, ADInstruments, Colorado Springs, CO, United States) and analyzed using LabChart 7 software (ADInstruments, Colorado Springs, CO, United States).

### Statistical Analysis

The results of Western blot analysis of sGCβ1 in the phrenic nerve and cGMP level measured with Elisa were statistically evaluated using One-Way ANOVA as well as the Unpaired *t*-test, and were expressed as means ± SEM. The level of significance ^∗^*p* < 0.05 was considered as significant. The number of PhMNs, motor axons in the PhN and NMJs at the diaphragm were statistically evaluated using the Unpaired *t*-test and were expressed as means ± SD (^****^p < 0.0001).

## Results

### Effects of PhN Ligation on sGCβ1 Changes in Phrenic Motor Pool

**FIGURE 1:**
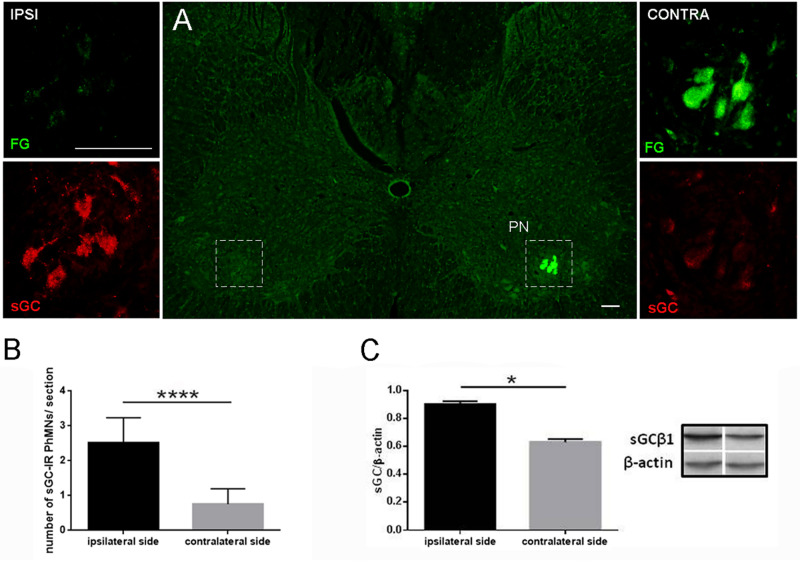
Soluble guanylyl cyclase β1 subunit immunoreactivity (sGCβ1-IR) in retrogradely labeled phrenic motoneurons and level of sGCβ1 protein in cervical spinal cord 8 days after unilateral PhN ligation **(A–C)**. Image of C4 spinal cord segment showing expression of retrogradely labeled phrenic motoneurons in contralateral phrenic nucleus (PN) 8 days after PhN ligation and bilateral injection of retrograde tracer Fluorogold (FG) into diaphragm **(A)**. Magnified area in box on right side shows intensive FG labeling and light sGCβ1-IR staining in motoneurons of contralateral PN. Intense expression of sGCβ1 and weak FG labeling is seen in phrenic motoneurons on ipsilateral side (left side). Number of retrogradely labeled sGCβ1-IR phrenic motoneurons/section in cervical segments (C3-C6) on contralateral and ipsilateral sides **(B)**. Level of sGCβ1 protein in ventral horn of C3-C6 segments after normalization to β-actin **(C)**. Data in **(B)** are mean values of five experiments ± SEM. Data in **(C)** are mean values of four experiments ± SEM. Results were statistically evaluated using Unpaired *t*-test; ^****^p< 0.0001 in **(B)**, ^∗^*p* < 0.05 in **(C)**. Scale bars = 1000 μm in **(A)**, 100 μm (insets in **A**).

Unilateral 8-day continuous ligation of the phrenic nerve was used to examine sGCβ1 in the lower bulbospinal pathway (phrenic motoneurons, phrenic nerves and NMJs at the diaphragm). As shown in Figure 1A, FG-IR phrenic motoneurons were clustered in groups of 3–5 motoneurons through the contralateral C3-C6 ventral horn. The sGCβ1 fluorescence signal was evident in FG-labeled motoneurons of the ipsilateral phrenic nucleus, while the sGCβ1 staining in the contralateral phrenic nucleus was substantially lower. Quantitative analysis revealed that the number of retrogradely labeled sGCβ1-IR phrenic motoneurons was significantly higher in the ipsilateral than in the contralateral phrenic nucleus (2.52 ± 0.71 vs. 0.76 ± 0.43; *p* < 0.0001) (Figure 1B). Similar results were achieved using WB analysis (Figure 1C). The level of sGCβ1 protein (normalized by the values of β-actin) was enhanced by 30% (0.903 ± 0.212 vs. 0.632 ± 0.020; *p* < 0.05) in the ipsilateral vs. contralateral ventral horn of C3-C5 segments. These results show that PhN ligation significantly modifies the expression of sGCβ1 in the phrenic motor microcircuits.

### Effect of PhN Ligation on sGCβ1 Changes in Phrenic Nerve

**FIGURE 2:**
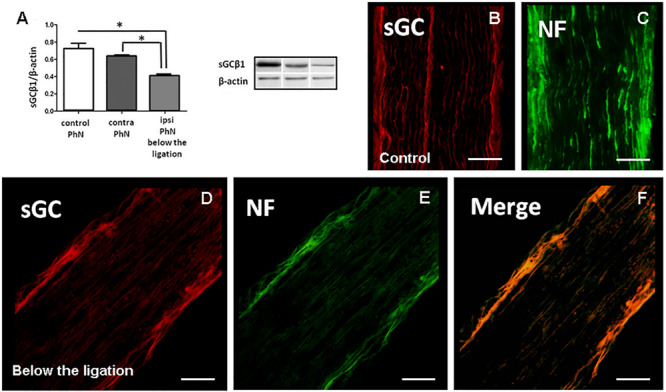
Soluble guanylyl cyclase β1 subunit immunoreactivity (sGC-IR), neurofilament immunoreactivity (NF-IR) and level of sGCβ1 protein in control phrenic nerve (PhN) in experimental animals 8 days after unilateral PhN ligation. Western blot analysis of sGCβ1 protein in PhN **(A)**. Longitudinal sections of PhN immunostained for sGC and neurofilaments (NF) in control **(B,C)**. sGC- and NF-IR in ipsilateral PhN below its ligation **(D,E)**. Merging of sGC- and NF-IR **(F)**. Level of sGCβ1 protein in **(A)** is normalized to β-actin. Data are mean values of four experiments ± SEM. Results were statistically evaluated using Unpaired *t*-test; *p < 0.05. Scale bars = 50 μm.

To study the effect of PhN ligation on the level of sGCβ1, the control, ipsilateral and contralateral samples of n. phrenicus were used for Western blot analysis (Figure 2A). The level of sGCβ1 protein (normalized by the values of β-actin) was 0.726 ± 0.060 in the control phrenic nerve. Eight days of continuous PhN ligation caused a significant decrease in sGCβ1 level in the nerve below its ligation (0.398 ± 0.006) compared to the contralateral nerve (*p* < 0.0001) or control nerve (*p* < 0.0006). WB analysis showed that the level of sGCβ1 protein was only slightly modified comparing control and contralateral phrenic nerves (*p* < 0.197). Immunolabeling with SMI-312 antibody (a specific marker for neurofilaments) was performed to visualize axons in the phrenic nerve. The neurofilament- and sGCβ1-IR axons were visible in the control group (Figures 2B,C). At the end of survival, the sGCβ1- and SMI-312-positivity decreased in axons below the nerve ligation (Figures 2D–F). These data indicate that PhN ligation decreased the level of sGCβ1 in motor axons, targeting the diaphragm innervation at the site of ligation.

### Degeneration of the Phrenic Nerve

**FIGURE 3:**
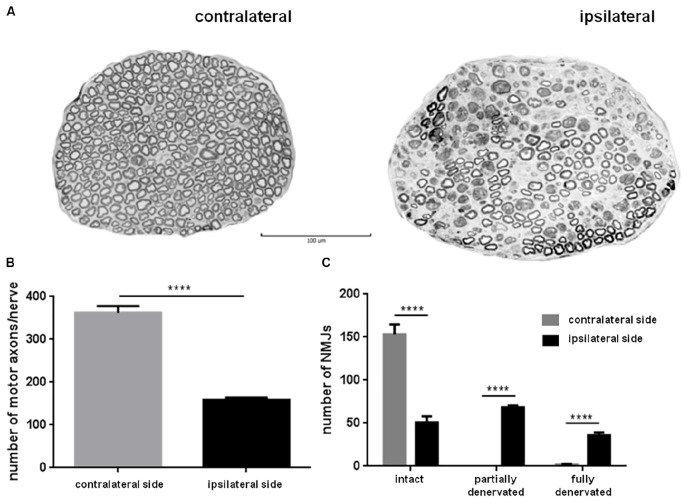
Eight days continuous phrenic nerve (PhN) ligation caused axonal degeneration in the ipsilateral PhN and abnormalities at neuromuscular junctions (NMJs) in the ipsilateral hemidiaphragm. Noticeable axonal degeneration in PhN below ligation point (ipsilateral) **(A)**. Quantitative analysis revealed significantly lower number of intact axons in ipsilateral than in contralateral PhN **(B)**. Numbers of intact, partially denervated and fully denervated (arrowhead) NMJs in ipsilateral vs. contralateral hemidiaphragm **(C)**. Data in **(B,C)** are mean values of five experiments ± SD. Results were statistically evaluated using Unpaired *t*-test; ****p< 0.0001. Scale bar = 100 μm.

We next examined anterograde degeneration of the phrenic nerve below the ligation point (Figures 3A,B). Axons of the contralateral phrenic nerve showed the typical morphological characteristics of intact myelinated axons. In the ipsilateral nerve, evident axonal loss was detected, and the tissue contained numerous disintegrated myelin structures typical for Wallerian degeneration. Axonal counts indicated massive loss of nerve fibers in the ipsilateral nerve (167.1 ± 0.060 vs. 357.8 ± 0.060 axons/nerve, *p* < 0.0001) 8 days after PhN ligation (Figure 3B).

### Axon Terminals in the Ipsilateral Hemidiaphragm Showed Reduced sGCβ1- and SV2-Immunoreactivity

**FIGURE 4:**
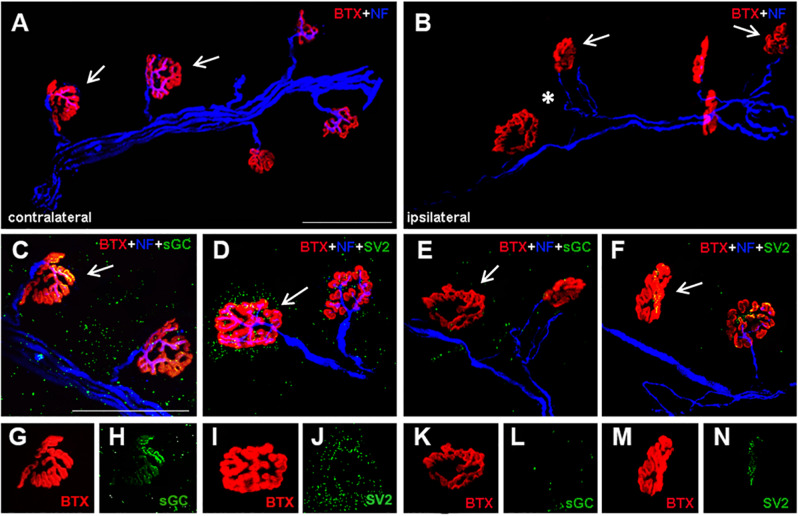
Representative confocal images showing morphological changes at diaphragm NMJs after unilateral PhN ligation. NMJs of diaphragm muscle were visualized *via* labeling post-synaptic acetylcholine receptors with rhodamine-conjugated α bungarotoxin (red), axons were labeled with anti-neurofilament antibody- SMI-312 (blue), soluble guanylyl cyclase β1 subunit immunoreactive terminals with sGCβ1 antibody (green) and presynaptic terminals with SV2 (green) **(A–N)**. All NMJs were intact in contralateral diaphragm (**A,C,D**, arrows), characterized by complete overlap of axons and presynaptic sGCβ1-IR terminals **(C)**, and axons and presynaptic vesicles **(D)** with postsynaptic acetylcholine receptors. Note dense accumulation of sGCβ1 stained puncta in contralateral diaphragm **(C,H)**. Ipsilateral hemidiaphragm shows NMJ abnormalities represented by partial (**B,E**, arrows) or complete denervation (**F,** arrows). Marked preterminal axon thinning is visible in ipsilateral diaphragm (**B,** asterisk). At NMJs in ipsilateral hemidiaphragm we detected almost complete reduction of sGCβ1- **(E,L)** and SV2-immunostained puncta **(F,N)**. Note single immunostaining from (**C,D**, arrows) in **(G,I)** – BTX, in **(H)** – sGC and in **(J)** – SV2. BTX **(K,M)**, sGC **(L),** and SV2 **(N)** in ipsilateral hemidiaphragm (single immunostaining from **E,F**, arrows). BTX- rhodamine-conjugated α bungarotoxin, NF-neurofilaments. sGC-soluble guanylyl cyclase. Scale bar = 75 μm.

Confocal imaging was used to visualize the sGCβ1-IR terminal boutons of the phrenic motoneurons at NMJs in the diaphragm (Figures 4A–C,E). As shown in Figures 4C,G,H, sGCβ1-IR puncta were visible in the contralateral hemidiaphragm inside the NMJs and beyond. Significant reduction in the area commonly occupied by the synaptic vesicles and/or sGCβ1 boutons over the endplates (Figures 4C,D,G–J) was seen in the ipsilateral diaphragm (Figures 4E,F,K–N). Similarly, marked reduction in sGCβ1 immunolabeled puncta was seen through the ipsilateral NMJs (Figures 4E,F,K–N). These results show that correlation exists between the loss of sGCβ1 protein and the loss of axons in the ipsilateral phrenic nerve, and the reduction in sGCβ1immunolabeled terminals in the ipsilateral NMJs.

### Morphological Alterations at Diaphragm NMJs After PhN Ligation

This was further supported by pathological alterations at the diaphragm NMJs. As shown in Figure 3C, differences in numbers of NMJs (intact, partially or fully denervated) between the contra- and ipsilateral hemidiaphragm were significant. Eight days after PhN ligation, the contralateral hemidiaphragm exhibited fully intact NMJs (99% ± 0.006). The number of intact NMJs was three-fold lower in the ipsilateral than the contralateral hemidiaphragm. Continuous PhN ligation induced a high percentage (67%) of NMJ abnormalities in the ipsilateral hemidiaphragm. These alterations were identified as partial (Figures 4B,E, arrows) or full denervation (Figure 4F, arrow). The percentage of NMJs exhibiting partial denervation was significantly greater (44%, *p* < 0.0001) compared to complete junction denervation (23%, *p* < 0.0001). However, the percentage of intact juctions declined to 33% (*p* < 0.0001) in the ipsilateral hemidiaphragm muscle (Figure 3C).

### cGMP Levels in the Diaphragm

ELISA assay was used to detect cGMP levels in the control diaphragm and in experimental hemidiaphragms 8 days after unilateral PhN ligation. Under physiological conditions, the level of cGMP was 36 ± 0.15 pmol/ml. Significant decrease in cGMP levels was observed in the contra- and ipsilateral diaphragm (*p* < 0.0001 vs. control) 8 days after PhN ligation. However, the decrease was nine – times stronger in the ipsilateral than the contralateral hemidiaphragm (0.7 ± 0.02 vs. 6.5 ± 0.01; *p* < 0.0001).

### EMG Activity in the Diaphragm

**FIGURE 5:**
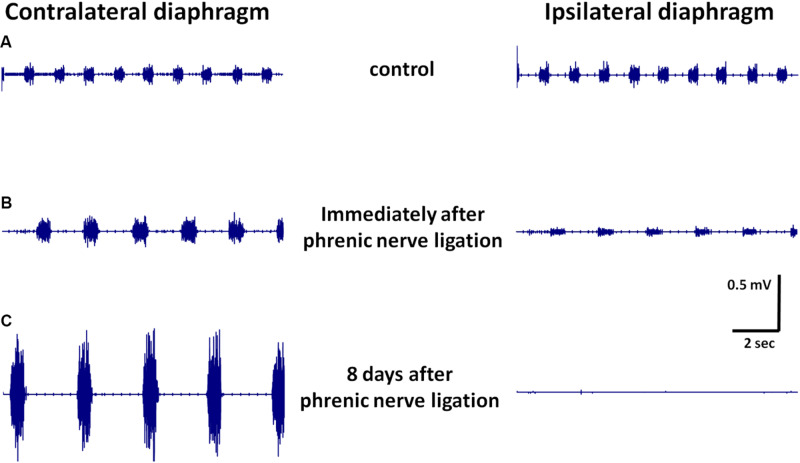
Representative EMG recordings from contralateral and ipsilateral hemidiaphragms in control animals **(A)**, immediately after phrenic nerve (PhN) ligation **(B)**, and 8 days after PhN ligation **(C)**.

Immediately after ligation the frequency of breathing decreased significantly by more than 40%, from ∼1.6 Hz in the control group to ∼0.9 Hz in the ligation group (Figures 5A,B). While there was no difference in breathing frequency on both sides of the diaphragm, the overall muscle activity differed dramatically. PhN ligation caused rapid and pronounced reduction in EMG activity amplitude on the ipsilateral side, while the amplitude of EMG on the contralateral side slightly increased (Figure 5B). Eight days after continuous PhN ligation, EMG on both sides of the diaphragm muscles changed markedly: on the ipsilateral side the residual activity disappeared, whereas on the contralateral side there was several-fold increase in EMG amplitude while breathing frequency remained unchanged (Figure 5C). This increase in EMG activity was due to an enormous increase in muscle fibers activity, but not to prolongation of that activity, since the duration of the rhythmic contractions did not change significantly.

## Discussion

Phrenic nerve injury is a well-established model, since the nerve ligation or transection deactivates descending excitatory drive from the phrenic motoneurons to the diaphragm. Within the framework of “Network Physiology” (Bashan et al., 2012; Bartsch and Ivanov, 2014; Bartsch et al., 2015; Lin et al., 2016) this study focuses on the network interactions between the central nervous system (cervical spinal cord) and the periphery (e.g., phrenic nerve and respiratory muscle). Experimental and clinical data have shown that PhN injury can have significant negative effects on diaphragm and lung, affecting their function. Recently published data revealed that the complete paralysis of the unilateral diaphragm could influence the loss of vital capacity and total lung capacity in the aged rats (Ding et al., 2020) but respiratory function parameters show compensation after 4 weeks in the young rats, and contralateral phrenic nerve transfer can enhance pulmonary function. In the present study we speculated, whether changes in the ipsilateral hemidiaphragm (e.g., loss of sGCβ1-IR nerve terminals, decreased level of cGMP, NMJs abnormalities and silencing of EMG activity) emerged from these interactions as an indicator of physiological state and function. To answer this fundamental question, immunohistochemical and histological staining, Western blotting, Elisa and electrophysiology were used. It is known, that the NO/sGC/cGMP signaling pathway plays a key role in regulation of a variety of vital function. However, its functional importance in the respiratory pathway (brainstem-phrenic motoneurons – phrenic nerve – diaphragm) still remains unclear. We have previously reported that the premotor bulbospinal respiratory pathway connecting the bulbar respiratory centers with the motoneurons of the phrenic nucleus in dog and rat is nitrergic (Marsala et al., 2002; Capkova et al., 2011; Hricová et al., 2012). In the present study we provide the first evidence that unilateral ligation of the phrenic nerve decreases the level of sGCβ1 in ipsilateral motor axons targeting the diaphragm innervation, and suggest that this effect is linked with loss of sGCβ1immunolabeled terminals at the ipsilateral MNJs. Subsequent remarkable unilateral decrease in cGMP level in the diaphragm and silencing of EMG activity can be attributed to the fact that sGC is involved in transmitter release at the NMJs via the sGC-cGMP pathway.

The phrenic nerves are generally considered as motor nerves whose primary function is to supply motor innervation to the diaphragm (Mantilla and Sieck, 2008). By means of a retrograde tracing technique we provide data showing direct projection from the hemidiaphragm to the contralateral FG-labeled phrenic motoneurons of the phrenic nucleus. Eight days of continuous PhN ligation caused significant increase in sGCβ1 (immunoreactivity and protein level) in the ipsilateral phrenic motor pool, but the nerve ligation reduced cGMP levels in the ipsilateral hemidiaphragm muscle. We suggest that continuous PhN ligation may lead to increased metabolic activity by ipsilateral phrenic α-motoneurons, normally expressing sGCβ1 at low level (Capkova et al., 2011). Recently published data indicate that 2 weeks of tetrodoxin (TTX) phrenic nerve blockade significantly increased both the total phrenic motoneuron surface area and the dendritic surface area (Mantilla et al., 2018). These authors suggest that ipsilateral phrenic motoneuron morphological adaptations are consistent with normalization of motoneuron excitability following prolonged alterations in motoneuron activity. Phrenic motoneuron structural plasticity is probably more dependent on motoneuron activity (or descending input) than muscle fiber activity. Furthermore, motoneurons have high metabolic activity associated with neurotransmission (Agar and Durham, 2003). They are relatively large cells, and therefore their permanent stimulation may induce unusually high influx of Ca^2+^ into these neurons (Greig et al., 2000; Vandenberghe et al., 2000; Van Den Bosch and Robberecht, 2000). Murphy et al. (1996) demonstrated that the primary excitatory drive for phrenic motoneurons is glutamatergic, probably acting via Ca^2+^-permeable, AMPA subtype glutamate receptors. As reported in previous studies (Saji and Miura, 1990; Marsala et al., 2002) nitrergic axons projecting from the dorsal respiratory group and/or rostral ventral respiratory group to the phrenic motoneurons release a signaling NO molecule as a co-transmitter with glutamate. We suggest that descending synaptic input of NO, which activates sGCβ1 in phrenic motoneurons (Capkova et al., 2011) and/or permanent blocking of NO-mediated cGMP synthesis in the hemidiaphragm after PhN ligation probably upregulates sGCβ1 in the ipsilateral phrenic motoneurons. The ability of motoneurons to handle calcium may also be important in determining their response to stimulation (Chao et al., 1997). Previous studies from our and other labs (Lips and Keller, 1998; Palecek et al., 1999; Lukáčová et al., 2012) have revealed a relatively low endogenous calcium-buffering capacity in motoneurons in comparison with other neuronal populations.

Phrenic motor neuron loss and subsequent diaphragm NMJ abnormalities are well described in various experimental models (Prakash et al., 1999; Nicaise et al., 2012, 2013; Fogarty et al., 2019), however, the correlation of these alterations with sGC transmission at the NMJs is lacking. Bashan et al. (2012) have shown that dramatic change in network structure with transition from one physiological state to another within a short time windows indicates a high flexibility in the interaction between physiological systems in response to change in physiological regulation. In the current study, we detected correlation between motor fiber damage, the reduction in sGCβ1-IR terminals and synaptic vesicles at the post synaptic acetylcholine receptors in the ipsilateral MNJs. Our in-depth diaphragm NMJ analysis demonstrated the phrenic axonal degeneration process, as many motor endplates were found to be fully (23%) or partially (44%) denervated as early as 8 days after PhN ligation. The pattern of diaphragm denervation has been studied in the central region of the diaphragm, particularly innervated by phrenic motor neurons located at the C5 level of the spinal cord (Laskowski and Sanes, 1987). In addition, we found significant loss of motor axons (∼47%) and changes in phrenic nerve morphology such as signs of Wallerian degeneration which affected morphological NMJ denervation and might have resulted in reduced spontaneous diaphragm EMG. We suggest that significant decrease in sGCβ1 level in the efferent arm of the respiratory pathway (diaphragmatic nerve) just below the nerve ligation site is also consistent with the cGMP level alterations in the diaphragm. Although we found marked reduction in cGMP levels in both the contra- and ipsilateral hemidiaphragm, the decrease was significantly greater (nine times) on the side of the ligation. Currently, the functional effects of unilateral 8-day continuous PhN ligation are unknown but significant effects can be inferred from recently published experimental study (Ding et al., 2020). Our results demonstrate that loss of motor axons and/or sGCβ1 in motor nerve terminal boutons after phrenic nerve ligation is consistent with alterations in synaptic transmission, the release of cGMP and endplate morphology in the ipsilateral hemidiaphragm.

As shown previously, most NO effects are mediated by sGC activation and subsequent cGMP formation (Reid et al., 1998; Koesling et al., 2005). cGMP influences the activity of at least three different targets, i.e., cGMP transduces the NO signal to the cGMP-regulated protein kinases, cGMP-activated phosphodiesterases (PDEs) and cGMP-gated ion channels (Southam and Garthwaite, 1993; Juilfs et al., 1999). As mentioned earlier, nNOS, cGMP and cGMP-dependent kinases are all concentrated at or near the NMJs (Kobzik et al., 1994; Koesling et al., 2004; Nickels et al., 2007). Reid et al. (1998) indicated that the rat diaphragm produces NO or NO-derivates which downregulate the contractile function via their effect on excitation-contraction coupling. Abraham et al. (1998) demonstrated that cGMP, which is present in most muscle fibers of the rat diaphragm, is primarily distributed in the subsarcolemmal region of individual fibers. It is important to emphasize that NO/sGC/cGMP signaling depends not only on cGMP formation, but it is also critically influenced by the activity of enzymes responsible for cGMP degradation (Koesling et al., 2005). In the diaphragm, PDEs degrade cGMP and terminate the cGMP-signal (Nickels et al., 2007). Our results support sGC-dependent cGMP signaling in the lower respiratory pathway, but the precise target of cGMP action in the diaphragm muscle still remains unclear.

Bartsch and Ivanov (2014) investigated the network of interactions between the brain, cardiac and respiratory system, and demonstrated that a network approach to physiological interactions is necessary to understand how modulations in the regulatory mechanism of individual systems translate into reorganization of physiological interactions across the organism. It is known that the function of inspiratory muscles is very important in the breathing process, because they are required to contract repetitively without interruption throughout life. Our results show remarkable unilateral silencing of EMG activity immediately after PhN ligation, and the failure of recovery of EMG activity in the ipsilateral diaphragm muscle 8 days after pathological continuous nerve ligation. These changes most likely depend on both the loss of ipsilateral phrenic axons (∼47%) and the degree of diaphragm denervation. We also show spontaneous and increased EMG activity in the contralateral hemidiaphragm. Such enhancement, recorded 8 days after unilateral PhN ligation, seems to be the result of increased muscle fiber activity. Mantilla and Sieck (2009) observed the absence of EMG activity in the ipsilateral diaphragm immediately and 3 days after C2 hemisection using diaphragm electrodes. They recorded progressive increase in the proportion of spontaneous recovery of EMG activity ispilaterally to C2 hemisection over time. Only 10% of unanesthetized animals displayed recovery at 7 days after hemisection. These findings are inconsistent with previously reported spontaneous ipsilateral increase in EMG activity. Vinit et al. (2008) reported that the ipsilateral phrenic nerve deactivated by lateral C2 SCI was spontaneously reactivated 7 days post-SCI. These authors also indicated that ipsilateral phrenic nerve reactivation was greater at 3 months compared with 7 days post-SCI, and that it was enhanced after contralateral phrenicotomy. There was gradual recovery of rhythmic diaphragm muscle activity ipsilaterally to cervical spinal cord injury over time, consistent with neuroplasticity and strengthening of spared, contralateral descending premotor input to the phrenic motoneurons (Mantilla et al., 2013). This suggests that spontaneous recovery of the respiratory pathway may depend on post-lesional time. After 8 days of hemidiaphragm paralysis induced by continuous PhN ligation, there was marked increase in sGCβ1 (immunoreactivity and protein level) in the phrenic motor pool, but the protein sGC level in the phrenic nerve below its ligation point and the cGMP level in the ipsilateral diaphragm were markedly decreased. These findings are consistent with alterations such as dramatic axonal loss below the nerve ligation site, reduction in sGCβ1-IR terminals/synaptic vesicles at the ipsilateral NMJs, significant morphological NMJ degeneration, and subsequent remarkable unilateral silencing of EMG activity.

Taken together, our results indicate the role of sGC/cGMP signaling in the lower respiratory pathway (phrenic motoneurons – phrenic nerve – diaphragm), although the complexity of respiratory circuits (e.g., relationship and coupling between the central nervous system and the periphery) requires further *in vivo* studies to clarify the functional contribution of this signaling pathway in respiratory neuroplasticity following cervical spinal cord and/or PhN injuries.

## Data Availability Statement

The datasets generated for this study are available on request to the corresponding author.

## Ethics Statement

This study was carried out in accordance with the principles of the Directive 2010/63/EU of the European Parliament and of the Council. The protocol was approved by the State Veterinary and Food Administration of the Slovak Republic (4434/16-221/3 and 715/19-221/3). All rights reserved under the supervision of the Ethical Council of the Institute of Neurobiology BMC SAS.

## Author Contributions

NL, LH, JG, and MM contributed to the study concept and design of the experiments. LH and SM performed the PhN ligation. LH and AK performed the Western Blotting and cGMP measurement using ELISA, Fluorogold was injected into the right diaphragm by JP. IV and ZD performed the PhN staining and quantitative analysis. LH, KB, MB, AS, and VL performed the immunohistochemical staining of spinal cord, diaphragm and phrenic nerve, and data analyses. JG and ŠP recorded the diaphragm EMG. LH and NL wrote the manuscript. All authors contributed to the manuscript revision, read and approved the submitted version.

## Conflict of Interest

SM was employed by the company Diagnostic Center of Pathology in Prešov, Alpha medical, s.r.o. The remaining authors declare that the research was conducted in the absence of any commercial or financial relationships that could be construed as a potential conflict of interest.

## Footnotes

1 https://home.saske.sk/~tomori/software/NeuroCounter

## Abbreviations

cGMP: cyclic guanosine monophosphate
EMG: electromyography
FG: fluorogold
GC: guanylyl cyclase
IR: immunoreactivity
NADPH: nicotinamide adenine dinucleotide phosphate
NMJ: neuromuscular junction
nNOS: neuronal nitric oxide synthase
NO: nitric oxide
PhMNs: phrenic motoneurons
PhN: phrenic nerve
sGC β 1: β 1 subunit of soluble guanylyl cyclase
sGC: soluble guanylyl cyclase.
